# Improved Photocatalytic Activity of Polysiloxane TiO_2_ Composites by Thermally Induced Nanoparticle Bulk Clustering and Dye Adsorption

**DOI:** 10.1021/acs.langmuir.1c01475

**Published:** 2021-08-17

**Authors:** Clara Chiappara, Giuseppe Arrabito, Vittorio Ferrara, Michelangelo Scopelliti, Giuseppe Sancataldo, Valeria Vetri, Delia Francesca Chillura Martino, Bruno Pignataro

**Affiliations:** † Department of Physics and Chemistry (DiFC) Emilio Segrè, University of Palermo, Building 17, V.le delle Scienze, Palermo 90128, Italy; ∥ National Interuniversity Consortium of Materials Science and Technology (INSTM), UdR of Palermo, Florence 50121, Italy; § Department of Biological, Chemical and Pharmaceutical Sciences and Technologies (STEBICEF), University of Palermo, Building 16, V.le delle Scienze, Palermo 90128, Italy

## Abstract

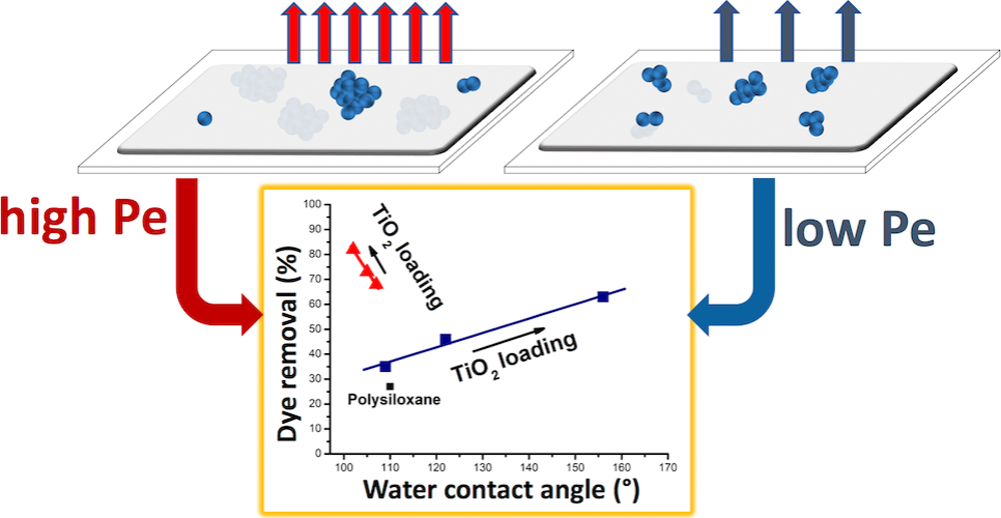

Fine control of nanoparticle clustering within polymeric matrices can be tuned to enhance the physicochemical properties of the resulting composites, which are governed by the interplay of nanoparticle surface segregation and bulk clustering. To this aim, out-of-equilibrium strategies can be leveraged to program the multiscale organization of such systems. Here, we present experimental results indicating that bulk assembly of highly photoactive clusters of titanium dioxide nanoparticles within an in situ synthesized polysiloxane matrix can be thermally tuned. Remarkably, the controlled nanoparticle clustering results in improved degradation photocatalytic performances of the material under 1 sun toward methylene blue. The resulting coatings, in particular the 35 wt % TiO_2_-loaded composites, show a photocatalytic degradation of about 80%, which was comparable to the equivalent amount of bare TiO_2_ and two-fold higher with respect to the corresponding composites not subjected to thermal treatment. These findings highlight the role of thermally induced bulk clustering in enhancing photoactive nanoparticle/polymer composite properties.

## 1 Introduction

The development of strategies for the reconfigurable assembly of polymer composites consisting of a mixture of polymers with nanoparticles^1^ or in general multicomponent mixtures is an emerging research topic.^2^ Among the nanoparticle systems, the favorable electronic and optical properties of metal oxide systems (e.g., TiO_2_, ZnO, AgO, and VO_2_) have fueled their widespread employment as active materials for different applications, such as photovoltaic cells,^3^ sensors,^4^ and photocatalytic degradation of pollutants.^5^ TiO_2_ nanoparticles have, in particular, received great interest for the development of various photovoltaic devices, from the dye-sensitized^6^ to the perovskite solar cells.^7^ They have been widely employed as outstanding photocatalyst systems,^8−10^ given their high efficiency, low cost, stability, and reusability, finding applications exploiting their antipollution and antibacterial properties.^11−15^ Also, 1D TiO_2_ nanomaterials have stimulated research interest in the energy and environmental fields, given their ability to produce confinement in the radial direction and the tunability of their bandgap via doping with rare-earth metals.^16^ However, the employment of bare TiO_2_ nanomaterials in photocatalysis is disadvantageous because of their ecotoxicity and the low absorption of visible light, which brings about low efficiency.^1,17^ For instance, the preparation of composites containing TiO_2_ nanoparticles within a polymeric material under selected conditions represents a potential solution to tackle this problem.^18^

In this context, selecting out-of-equilibrium conditions for the assembly of nanoparticle/polymer composites can improve the resulting physicochemical properties compared to those obtainable under thermodynamic control.^19^ The success in this approach critically depends on the careful selection and tuning of physicochemical parameters compatible with polymer-based materials including light,^20^ temperature,^21^ magnetic fields,^22^ and suitable chemical cues.^23^ In the context of nanoparticle/polymer composites, temperature is an easily controllable physical parameter that can be conveniently and readily controlled to govern the formation of composite architectures by the interplay of polymer stratification, particle mixing^24^ into the polymer (segregation, sedimentation, and diffusion), and evaporation of the liquid dispersing medium.^25^ Indeed, the perturbations imposed by the solvent evaporation can guide the nanoparticle assembly dynamics, in a balance between thermodynamic phase separation and nanoparticle dispersion within the polymer matrix.^25^ It has been recently demonstrated that the polymer size and stiffness can tune the dispersion, the aggregation, and the morphology of the nanoparticles in thermally controlled conditions.^25^ These polymeric features are used to specifically tune geometry and size of the nanoparticle cluster but not their surface segregation, which would concur to reduce the total surface energy of the composite. To this regard, it is known that the nanoparticle clustering can lead to stratification at the surface or in the bulk of the material due to thermophoresis or diffusiophoresis, which are dependent on the nanoparticle size and the solvent evaporation rate.^25^ In general, the competition between evaporation and diffusion can be expressed by the Péclet number, *Pe* = *Hv*/*D*, *H* being the thickness of the interface where evaporation occurs, *D* is the diffusion coefficient of the particles, and *v* is the receding speed of the interface.^26^ For *Pe* ≫ 1, the particles are segregated at the liquid/vapor interface since their diffusion is slower in comparison to the reduction of the interface. In the case of *Pe* ≪ 1, the particle diffusion is faster than the motion of the interface, and the particles remain uniformly distributed in the drying film.

Among many examples of dispersing materials for TiO_2_ nanoparticles, a special mention needs to be given to diatomite,^27^ hydrophobic polysiloxane polymers,^28^ fluorinated polysiloxane,^29^ and nylon-6,^30^ all resulting in an optimal balance between photodegradation efficiency and mechanical stability. Specifically, polysiloxanes represent a convenient choice, given their optimal mechanical properties,^31^ thermal stability,^32^ optical transparency,^33^ and tunable surface hydrophobicity.^34,35^ For example, Ding and coworkers^29^ have shown the possibility of realizing superhydrophobic TiO_2_ composites with fluorinated polysiloxanes, reporting the retaining of photocatalytic properties under UV light irradiation. The above reported polymer/TiO_2_ nanoparticle composites are typically obtained at conditions in which the evaporation dynamics is controlled by particle diffusion in the composite film (i.e., *Pe* ≪ 1), resulting in their distribution between the bulk of the material and the air/solid interface. The result of this process is surface exposure of TiO_2_ nanoparticle clusters covered by polymer molecules due to the minimization of the surface energy under thermodynamic control.^29^ This ultimately leads to coatings with significant superhydrophobic properties.^23^

A crucial aspect governing the photocatalytic properties of the resulting TiO_2_ nanoparticle/polymer composites is constituted by the TiO_2_ nanoparticle cluster sizes and connectivity. A recent report has shown that increasing TiO_2_ nanoparticle cluster size improves their stability without affecting the photocatalytic activity.^30^ In this context, research on colloidal TiO_2_ films has shown the possibility of controlling the particle aggregation by solvent removal to produce nanoparticle networks containing a high abundance of functional interfaces.^36^ Another approach employs low-temperature TiO_2_ nanoparticle sintering (100 °C) to obtain an electrically connected nanoparticle network, which was shown as a good dye-sensitized anode in the field of photoelectrochemical cells.^37^ It could be expected that increasing cluster size might improve the photocatalytic properties of photoactive nanoparticles, as suggested in a recent report from Nandiyanto et al.,^38^ which has demonstrated the beneficial effect on the increased diffusion time of the light-generated carriers by increasing the size of monoclinic WO_3_ microparticles. However, to our knowledge, it is unknown whether thermally induced TiO_2_ nanoparticle clustering effects within polymeric matrices might tune the photocatalytic properties of the resulting composite.

This work reports on an innovative TiO_2_ nanoparticle bulk clustering approach within an in situ produced polysiloxane matrix. A suitable thermal treatment on the TiO_2_ nanoparticle/polysiloxane mixture at the solvent boiling temperature (80 °C, 1 h) allows control of the evaporation dynamics of such a system, favoring TiO_2_ nanoparticle partial sintering, bulk dispersion, and clustering, ultimately maintaining the surface wetting properties of the polymeric system. The resulting TiO_2_ nanoparticle/polysiloxane composite is cast onto glass surfaces to produce compact thick coatings showing a two-fold enhancement of the photocatalytic activity with respect to the system prepared without thermal treatments.

## 2 Experimental Section

### 2.1 Materials

Titanium(IV) oxide nanoparticles (Sigma Aldrich; primary size, 21 nm; Degussa P25), ethyl acetate (EtOAc, Fisher, 99.5%; MW, 88.106 g/mol), hydride-terminated poly(dimethylsiloxane) (PDHS; Sigma Aldrich; Mn, ∼17,500 g/mol), 1*H*,1*H*,2*H*,2*H*-perfluorooctyltriethoxysilane (POTS; Sigma Aldrich; Mn, 510.36 g/mol), a Karstedt catalyst (platinum(0)-1,3-divinyl-1,1,3,3-tetramethyldisiloxane complex solution, Sigma Aldrich; MW, 381.48 g/mol), and methylene blue (MB; Sigma Aldrich; MW, 319.85 g/mol) were used.

### 2.2 PDHS-POTS Coating Preparation

**Scheme 1:**
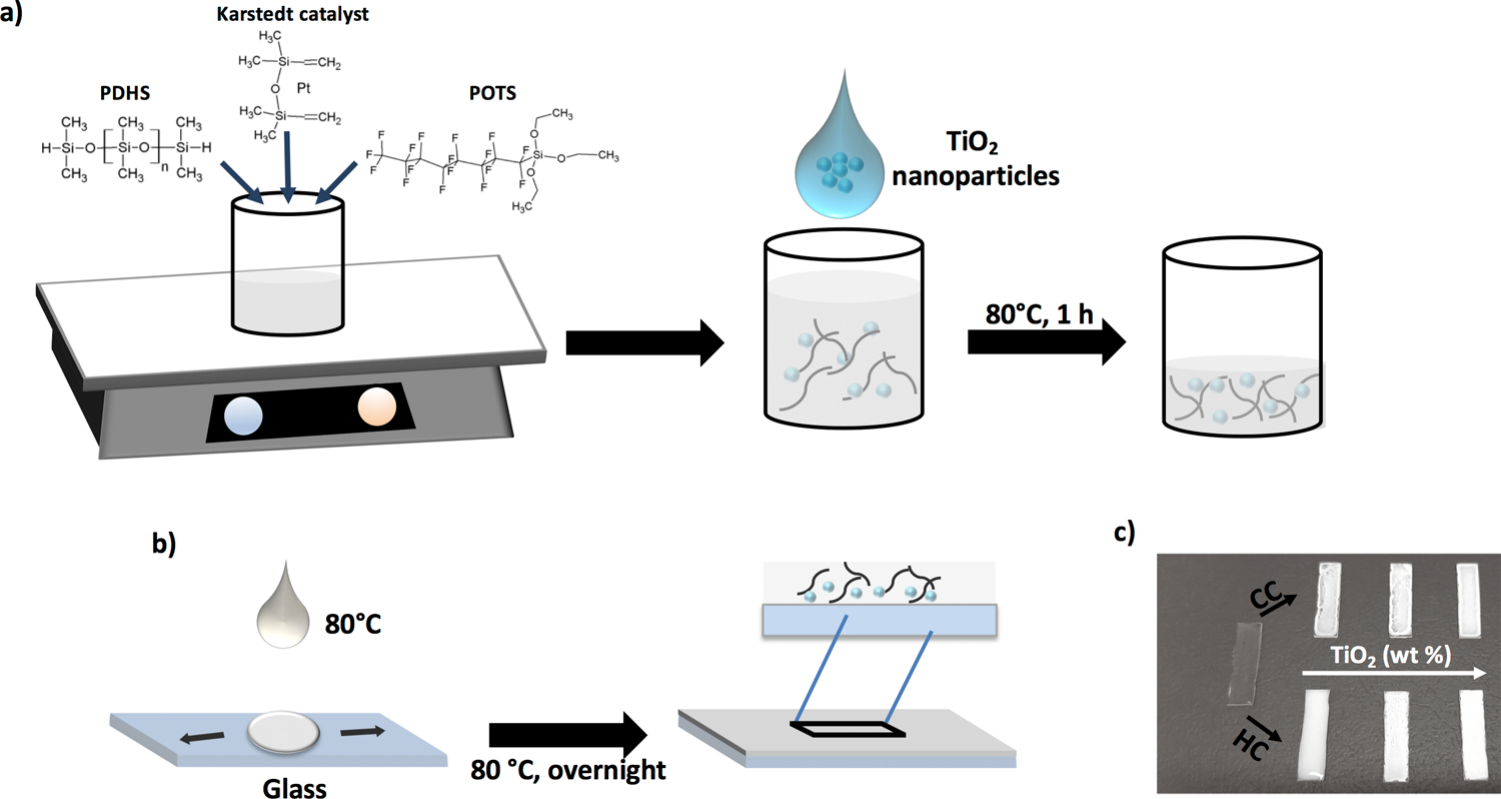
The Strategy Employed for the Realization of Composite Coatings on Glass Surfaces by the Employment of Thermal Treatments (a) The mixture containing PDHS-POTS and TiO_2_ nanoparticles is heated at 80 °C for 1 h, allowing partial removal of EtOAc. (b) While still at 80 °C, the nanoparticle/polymer mixture is drop-cast onto a glass slide for producing a thick coating, defined as heated coating (HC), until complete removal of EtOAc by drying the composite film at 80 °C overnight. In the case of control coating (CC), the same mixture is deposited at room temperature and then subjected to the same drying conditions. (c). Photograph of the resulting HC and CC systems at different TiO_2_ nanoparticle concentrations (5, 15, and 35 wt %).

In a plastic beaker, 9 g of PDHS was added to solution containing 0.2 g of POTS in 50 mL of EtOAc (see Scheme 1a). Then, the Karstedt catalyst ([Pt]/[Si–H] = 4 × 10^–6^) was added into the above mixture. The resultant sol mixture was stirred for 48 h at room temperature until the PDHS-POTS polymer was obtained, following the procedure shown in the previous reports.^29^ The coating was prepared by drop-casting of the PDHS-POTS resultant polymer sol onto glass and kept at 80 °C.

### 2.3 Composite Coating Preparation

The TiO_2_-polysiloxane composite coatings (7 mm × 20 mm) were prepared at different amounts of TiO_2_ (5, 15, and 35 wt % corresponding to 1.2, 3.7, and 8.7 mg in 65 mg of the PDHS-POTS polymer), following two different procedures, which resulted in the heated coating (HC) and control coating (CC) systems (see Scheme 1b,c). In the case of HC, the sol was realized by adding to the PDHS-POTS polymer the above reported amounts of TiO_2_ dispersed in 1 mL of EtOAc. The resulting sol was subjected to thermal incubation (80 °C, for 1 h), slowly removing EtOAc to obtain a final volume of 0.4 mL. The resulting mixture, kept at 80 °C, was drop-cast onto glass and maintained at 80 °C until complete drying. In the case of CC, the sol was realized by adding to the PDHS-POTS polymer the above reported amounts of TiO_2_ dispersed in 0.4 mL of EtOAc and was rapidly drop-cast onto glass at ambient temperature and then kept at 80 °C, until complete drying. In addition, a TiO_2_ coating on glass (containing the same amount of nanoparticles as the 35 wt % samples) was prepared by a TiO_2_ dispersion in EtOAc and kept at 80 °C until complete drying. The temperature used for the preparation of these coatings (80 °C) was chosen to allow for controlled evaporation of the solvent avoiding cracks on the coatings, as observed for higher annealing temperatures (such as 100 °C).

### 2.4 Characterization of Composite Coatings

The polymer synthesis and the resulting composite chemical properties were characterized by ATR-FTIR with an FTIR Bruker Vertex 70v spectrophotometer with an accessory platinum ATR, with 2 cm^–1^ steps and 60 scans in the acquisition range 4000–500 cm^–1^ at 2 hPa. A baseline correction of the scattering was performed using OPUS 7.5 software. The surface chemical composition of the coatings was investigated by XPS using an ULVAC-PHI 5000 Versa Probe II scanning XPS microprobe, an Al Kα source (1486.6 eV), a 128-channel hemispherical analyzer, and FAT mode. Static contact angle (CA) measurements (10 μL drops) were performed using three liquids (ultrapure water, glycerol, and tricresyl phosphate, TCP) to investigate the hydrophobicity of the composites and to quantify the surface free energy by the van Oss–Chaudhury–Good method.^39^ The CA values were calculated as an average of five measurements. The surface morphology of the coatings was observed with scanning electron microscopy (FEI Versa 3D). The samples were placed on stubs and sputter-coated with gold before SEM imaging, at an accelerated voltage of 10 kV. The cluster organization of the composites was evaluated by employing Otsu’s algorithm grain analysis implemented in Gwyddion^40^ (2.53 version). The two- and three-dimensional bulk structure of the composites was characterized using a Leica TCS SP5 fluorescence confocal laser scanning microscope (CLSM), using a 63X-1.40 NA oil objective (Leica Microsystems, Germany). The composites were marked with the fluorescent dye Nile red by drop-casting 500 μL of the aqueous dye (0.5 μM) on the coating surface for 30 min before washing. Imaging was performed using λ_ex_ = 500 nm (“white light laser”, Leica Microsystems), and emitted and scattered signals were detected in the range λ_em_ = 520–700 nm. The three-dimensional reconstructions of the composites were obtained from 60 images (1024 × 1024 pixels) per stack acquired along the *z*-axis with a 0.5 μm step for 30 μm. Data were analyzed using the ImageJ software 1.52p version.

### 2.5 Photocatalytic Activity Measurement

The photocatalytic activity of coatings was evaluated in terms of photodegradation of MB in aqueous solution (3 mL, 25 μM). Irradiation was carried out using a solar simulator (ABET Solar Simulator Model 10500) at 1 sun. The distance between the sample and the solar simulator was set using a calibration cell through a tester (1 sun corresponded to 100 mV). Before light irradiation, the coatings were soaked into the MB solution and kept in the dark for 1 h to allow adsorption–desorption equilibrium. Irradiation effects were evaluated at 30 min intervals by analyzing changes in the absorption spectra of the MB solution. Spectra were acquired using a UV–vis spectrophotometer (Specord S600), in the range 200–800 nm, with a wavelength accuracy of ±0.3 nm (reported by the producer). Intensity measurements at 664 nm were used to quantify spectral changes. The reusability of the PDHS-POTS HC and CC systems at 35 wt % TiO_2_ was tested by repeating the cycles of MB solution degradation on the same sample up to three times, in the same conditions describe above. In between each photocatalytic test, the sample was washed with ultrapure water (Direct Q-UV filtration system, 18.2 MΩ cm). The reproducibility of the PDHS-POTS HC and CC systems at 35 wt % TiO_2_ was investigated by replicated photocatalysis experiments on three different samples.

## 3 Results and Discussion

### 3.1 IR Chemical Characterization of the PDHS-POTS Systems

**Figure 1:**
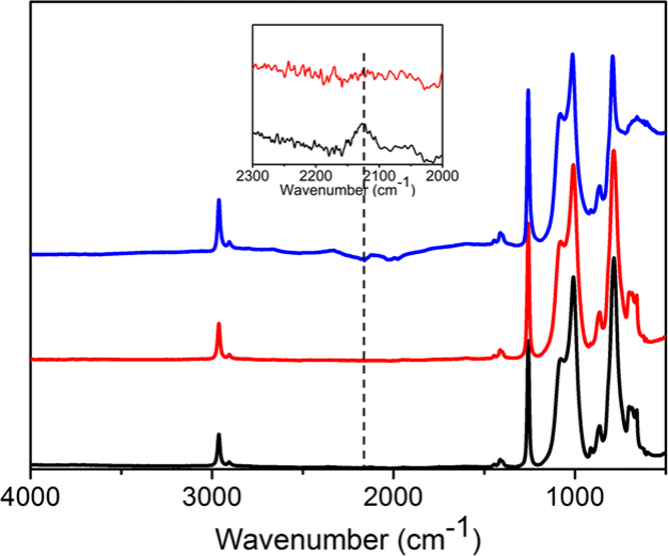
ATR-FTIR spectra of (black) PDHS, (red) PDHS-POTS, and (blue) PDHS-POTS/TiO_2_ (35 wt %) HC (blue) coatings. The reported inset shows the spectral region in the interval 2300–2000 cm^–1^, reporting on the Si–H bond stretching signal present in PDHS (black) and the disappearance of the signal in PDHS-POTS (red).

The outcome of the reaction between POTS and PDHS was shown by analyzing the ATR-FTIR spectra acquired on the coatings after annealing at 80 °C in the spectral region between 4000 and 500 cm^–1^ (see Figure 1). The spectra were acquired from bare PDHS, PDHS-POTS, and PDHS-POTS/TiO_2_ (35 wt %) HC coatings.

The success of the hydrosilylation reactions and dehydro-coupling of PDHS with POTS (already known^41^) is demonstrated by the disappearance of the characteristic signal of the stretching of the Si–H bond at 2126 cm^–1^, which is present only in the PDHS chemical structure. The Si–H bond signal is weak, being the contribution of two bonds in a high molecular weight polymer (17,500 g/mol). It is also possible to detect other diagnostic IR-active modes relevant for characterizing the system. In particular, the bands at 1089 and 1010 cm^–1^ can be assigned to the asymmetric stretching of Si–O–Si,^42^ while that at 783 cm^–1^ is due to the symmetrical one of the Si–O bond.^27,43^ The peak at 1253 cm^–1^ can be ascribed to the Si–CH_3_ groups,^44^ while the 500–700 cm^–1^ wide band present in the composite can be assigned to the vibration of the Si–O–Ti and Ti–O–Ti bonds.^27,45^ These data highlight the presence of all the expected functional groups in the obtained composites.

### 3.2 Physicochemical Characterization of the Coating Surfaces

**Figure 2:**
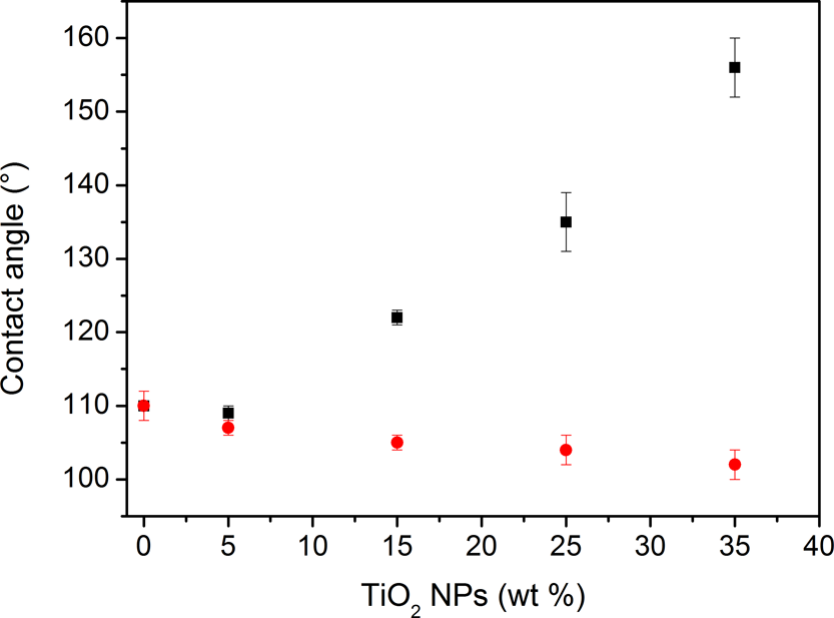
Variation of water CA as a function of the TiO_2_ nanoparticle amount (from 0 to 35 wt %) on PDHS-POTS/TiO_2_ HC (red dots) and PDHS-POTS/TiO_2_ CC (black squares) systems. Each value is the average of five different measurements.

The surface chemistry of the two different composites was then investigated by static water contact angle (CA) measurements, permitting evaluation of the effect of TiO_2_ nanoparticle clustering on the polymer film surface free energy (SFE). The water CA was first studied as a function of the TiO_2_ nanoparticle loading (0–35 wt %) in the polymer structure, as reported in Figure 2. Importantly, measurements on HC systems reveal a slight decrease in the contact angle at increasing TiO_2_ nanoparticle amounts, whereas a significant increase in the contact angle is observed in CC samples. The measured CA appears to be not significantly affected by the TiO_2_ presence being about 110 ± 2° at TiO_2_ concentrations below 5%. It is worth noting that the CC samples at a higher concentration of TiO_2_ (35 wt %) are characterized by a water CA of about 156 ± 4°, which is typical of superhydrophobic composites.^29^

As reported in Table 1, for the PDHS-POTS and the PDHS-POTS/TiO_2_ (35 wt %) HC system, the SFE values are similar (both ≈ 25 mJ/m^2^). The SFE components for both systems remarked the predominant hydrophobic character of the surface that could be ascribed to the fact that the TiO_2_ clusters mainly segregate in the bulk phase of the polymer matrix, being only partially segregated at the surface of the composite. A slightly higher γ^+^ component is detected for the HC system with respect to the bare polymer, possibly due to the surface segregation of apparently hydrophilic TiO_2_ clusters, which finally result in a higher surface wettability/hydrophilicity, confirmed by the slightly lower θ_w_ measured for HC with respect to the bare polymer. The SFE calculated for PDHS-POTS/TiO_2_ (35 wt %) CC was equal to 20 mJ/m^2^, which is lower than that of the bare polymer or HC system, revealing an increased hydrophobicity. In addition, an increased basic component was calculated, which could be ascribed to the presence of surface-segregated TiO_2_ clusters covered by PDHS-POTS.

**Table 1:** CA Values (Expressed in Degrees) Measured with Water (θ_w_), Glycerol (θ_g_), and TCP (θ_TCP_) Droplets on the Three Reported Solid Surfaces Allowing the Estimation of SFEs and the Lifshitz/van der Waals (γ^LW^), Basic (γ^–^), and Acidic (γ^+^) Components^a^

coatings	θ_w_	θ_g_	θ_TCP_	SFE	γ^LW^	γ^–^	γ^+^
PDHS-POTS	110 ± 2	115 ± 1	68 ± 1	25 ± 1	19.2 ± 1	2.0 ± 0.1	3.5 ± 0.1
PDHS-POTS/TiO_2_ (35 wt %) HC	102 ± 2	108 ± 4	66 ± 1	24 ± 1	20.1 ± 1	1.1 ± 0.1	4.4 ± 0.2
PDHS-POTS/TiO_2_ (35 wt %) CC	156 ± 4	153 ± 1	72 ± 5	20 ± 2	17.7 ± 1	7.0 ± 0.5	0.2 ± 0.1

a The values of SFE, γ^LW^, γ^–^, and γ^+^ are all expressed in mJ/m^2^. The reported CA values are an average value of four different measurements performed on each coating.

**Figure 3:**
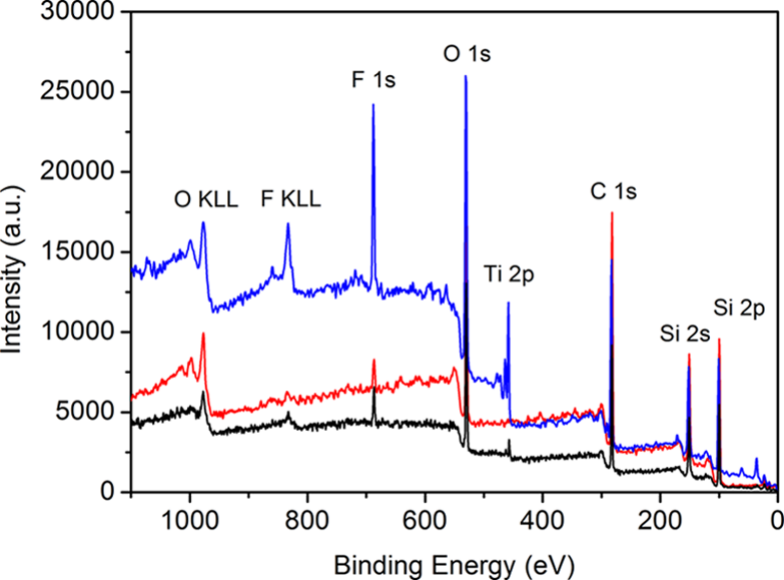
XPS survey spectra of (red) PDHS-POTS coating, (black) PDHS-POTS/TiO_2_ (35 wt %) HC, and (blue) PDHS-POTS/TiO_2_ (35 wt %) CC.

The surface chemistry of the composites was also investigated by XPS quantitative analysis. Figure 3 reports on the XPS survey spectra of the three different systems, i.e., PDHS-POTS, PDHS-POTS/TiO_2_ (35 wt %) HC, and PDHS-POTS/TiO_2_ (35 wt %) CC. The resulting quantitative analysis on the three samples (see Table 2 reporting the elemental composition analysis on the composites expressed as atomic percentages) supports the SFE data previously obtained. The data reported in Table 2 show that the CC (35 wt % TiO_2_ ) is characterized by a high fluorine and titanium content, suggesting the formation of TiO_2_ nanoparticle clusters covered by the polymer on the surface of the CC system, with the possible orientation of the fluorinated groups that make superhydrophobic films, as already known by superhydrophobic TiO_2_-polysiloxane composites.^29^ In the case of HC (35 wt % TiO_2_), both the fluorine and titanium contents are significantly lower than the corresponding CC system, confirming the low surface segregation of the TiO_2_ clusters accordingly with a lower surface hydrophobicity. The fluorine content of HC is still higher than the corresponding one of the bare polymer, as a likely consequence of the minimal presence of surface-segregated TiO_2_ clusters.

**Table 2:** Atomic Percentage Values (at. %) Obtained from the XPS Surface Analysis of the Investigated Coatings

coatings	C 1s	O 1s	F 1s	Si 2p	Ti 2p
PDHS-POTS	48.84	27.08	1.01	23.48	
PDHS-POTS/TiO_2_ (35 wt %) HC	46.90	27.30	2.54	22.86	0.39
PDHS-POTS/TiO_2_ (35 wt %) CC	35.59	29.56	12.60	18.80	3.45

**Figure 4:**
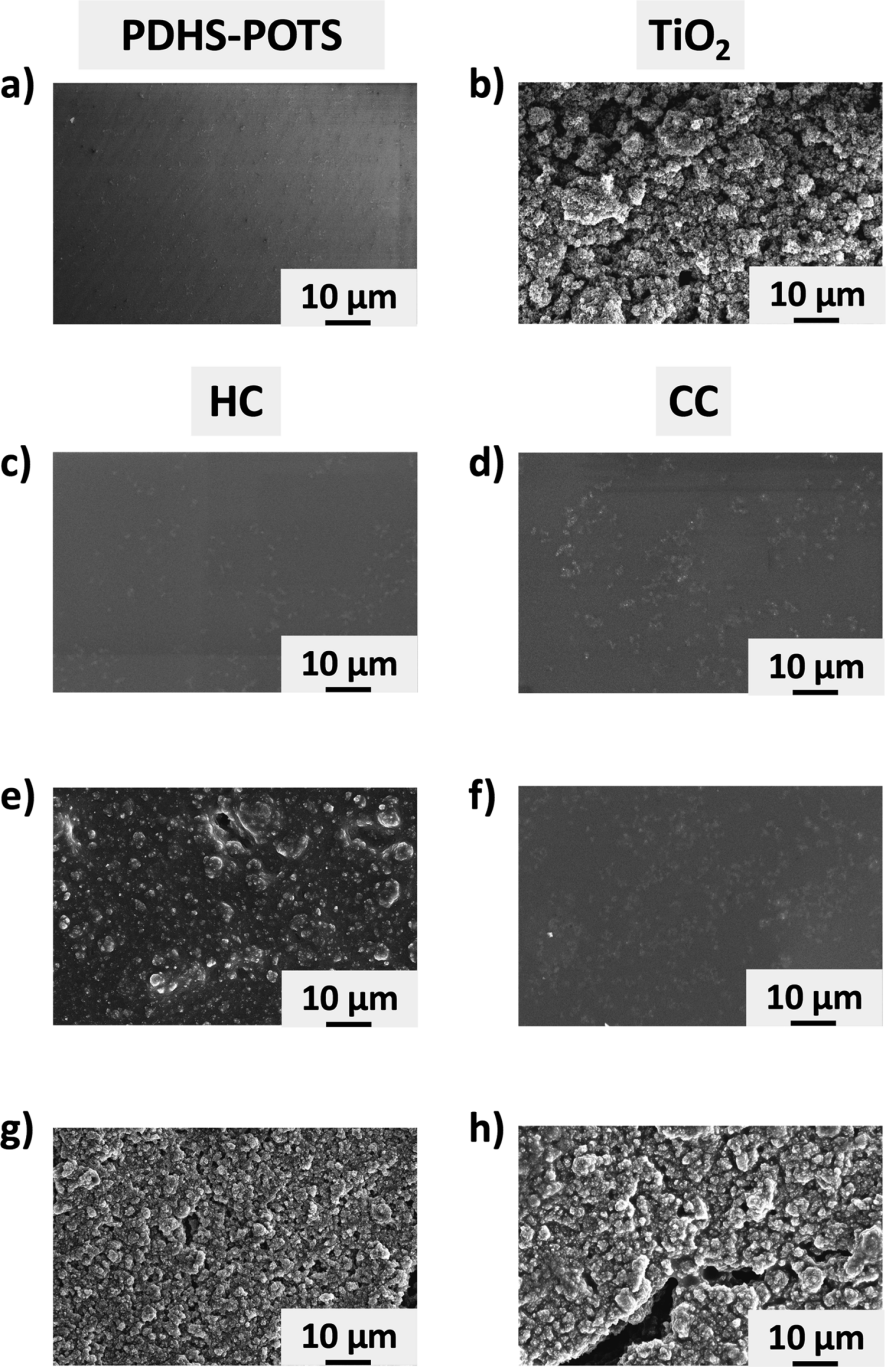
SEM morphological investigation of (a) PDHS-POTS coating, (b) TiO_2_ coating, (c) PDHS-POTS/TiO_2_ (5 wt %) HC, (d) PDHS-POTS/TiO_2_ (5 wt %) CC, (e) PDHS-POTS/TiO_2_ (15 wt %) HC, (f) PDHS-POTS/TiO_2_ (15 wt %) CC, (g) PDHS-POTS/TiO_2_ (35 wt %) HC, and (h) PDHS-POTS/TiO_2_ (35 wt %) CC.

The striking differences in the surface chemistry of the CC and HC were then confirmed by surface and bulk morphological characterizations. The surface morphologies of the HC and CC systems were investigated by SEM as a function of the different TiO_2_ loadings and compared to those of the PDHS-POTS (Figure 4a) and TiO_2_ coatings (Figure 4b).

The surface of the PDHS-POTS/TiO_2_ (5 wt %) HC (Figure 4c) appears to be covered by micrometric clusters, which are smaller in comparison to the CC prepared under the same amount of TiO_2_ (Figure 4d). Interestingly, the PDHS-POTS/TiO_2_ (15 wt %) HC system is characterized by significantly larger clusters (Figure 4e) with respect to those observed for the PDHS-POTS/TiO_2_ (5 wt %) CC system (Figure 4f). Finally, the morphologies of the PDHS-POTS/TiO_2_ (35 wt %) HC and CC systems (Figure 4 panels g and h, respectively) are both dominated by large clusters, which resemble the TiO_2_ coating surface. The SEM characterization of the TiO_2_ clusters collected at a higher magnification highlights their globular morphology (see Figure S1). As expected, the total projected area by the clusters increases with the TiO_2_ amount in both the two composites (see Figure S2). The same trend is observed from the arithmetic average roughness evaluated from the SEM images (see Figure S3). The two analyses confirm the differences observed comparing HC and CC systems. Indeed, whereas in the PDHS-POTS/TiO_2_ (5 wt %) case, the HC system has a lower clustered area and lower roughness with respect to CC, for the higher TiO_2_ loadings (15 and 35 wt %), the opposite scenario is observed. The surprising differences in the CC and HC surface structural organization observed by SEM characterizations agree well with the diverging trends of surface hydrophobicity obtained from the previously reported CA measurements of the PDHS/POTS systems as a function of the TiO_2_ loading. Accordingly, the TiO_2_ clustering effects clearly affect the surface hydrophobicity of the resulting composite material.

### 3.3 3D Characterization of the Composite Materials

**Figure 5:**
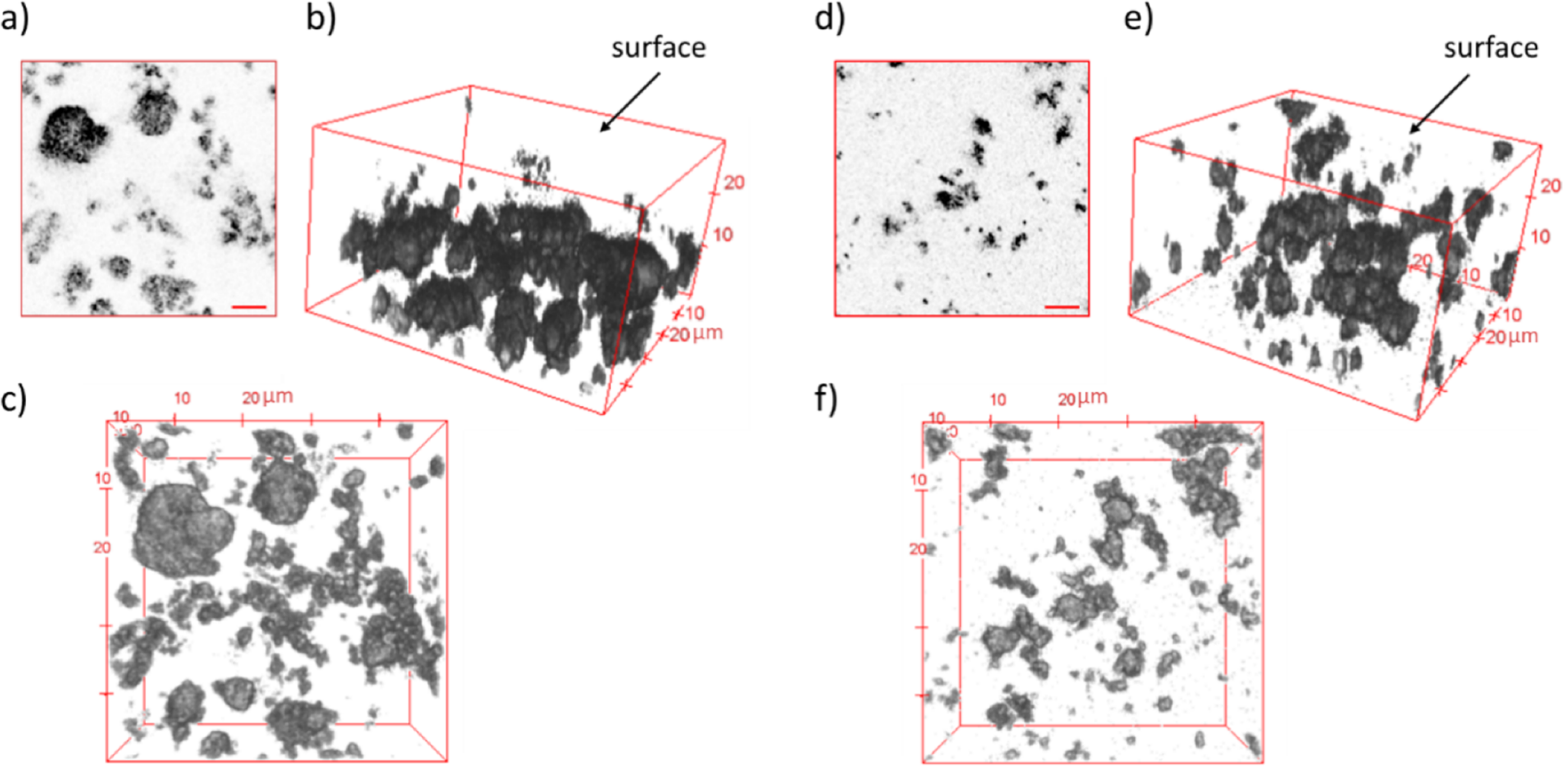
CLSM imaging of PDHS-POTS/TiO_2_ (5 wt %) HC (a–c) and CC (d–f). Representative 1024 × 1024 sections measured at a height of 6 μm of PDHS-POTS/TiO_2_ (5 wt %) HC (a) and CC (d) (scale bars, 10 μm); 3D reconstructions (analysis depth of 30 μm from the surface to the bulk) of PDHS-POTS/TiO_2_ (5 wt %) HC ((b,c) side view and top view, respectively) and CC ((e,f) side view and top view, respectively).

In order to shed light on the three-dimensional bulk organization of the systems, the relevant structural features were investigated using a CLSM, which shows different microscale morphologies for the HC and CC systems containing TiO_2_ at 5 wt %. Images (50 μm × 50 μm) were acquired along the *z*-axis up to 30 μm depths, using 0.5 μm steps. Representative results of the measurements are reported in Figure 5. In panel (a), a section acquired at a 6 μm quote is reported for HC, together with 3D reconstructions at different perspectives (b,c). Similarly, in panel (d), a section at a 6 μm quote is reported for CC, together with 3D reconstructions using different perspectives (e,f). In the images, the higher intensity is attributed to TiO_2_ nanoparticle clusters (gray), diffused signals (light gray) to the polymeric component. The acquired signal is mainly dominated by scattering components (including Raman scattering and diffuse reflectance), and low Nile red fluorescence was observed. The reported measurements highlight that both systems are characterized by TiO_2_ nanoparticle clusters dispersed in the PDHS-POTS polymeric matrix. Notably, different sample preparations lead to different composite bulk architectures. The HC sample is characterized by large TiO_2_ nanoparticle clusters (gray) mainly localized in bulk, thus being excluded from the surface. A completely different scenario was observed in the case of the CC sample, reported in Figure 5d–f. In this case, smaller and more homogeneously distributed TiO_2_ clusters are distributed along the *z*-axis, without significant differences in the cluster dispersion between the bulk and the solid/air interface.

The investigations on the HC and CC systems reveal the critical differences in morphology and hydrophobicity induced by the TiO_2_ clustering and surface vs bulk segregation. In the case of CC, as expected, the TiO_2_ nanoparticle clusters are both bulk-dispersed and surface-segregated. The latter are covered by PDHS-POTS, resulting in a highly hydrophobic surface, with a slightly higher basic component regarding the bare polymer surface. This can be due to the presence of exposed oxygen atoms from the TiO_2_ nanoparticle clusters. Accordingly, the XPS analysis shows high titanium and fluoride contents, which further support the surface composition of this system, in agreement with the composition features of superhydrophobic TiO_2_/polymer coatings. Differently, HC is characterized by a bulk organization of larger TiO_2_ nanoparticle clusters, whereas the surface is characterized by few smaller TiO_2_ clusters and few PDHS-POTS molecules, resulting in a surprising increase in hydrophilicity with respect to the bare polymer. It is known that the TiO_2_ surface is amphotheric^46^ and that in particular, P25 is characterized by Lewis acid sites (Ti^4+^ ions) and basic sites (O^2–^ ions), which are also responsible for the photocatalytic activities of this system.^47^ Accordingly, such acid properties are slightly increased only in the case of the HC system and are likely responsible for the improved wettability properties. The above presented investigations on the HC and CC systems revealed significant differences in the surface and bulk morphologies and hydrophobic behaviors, which are likely expected to modify the resulting photocatalytic properties.

### 3.4 Analysis of Photocatalytic Activity

**Figure 6:**
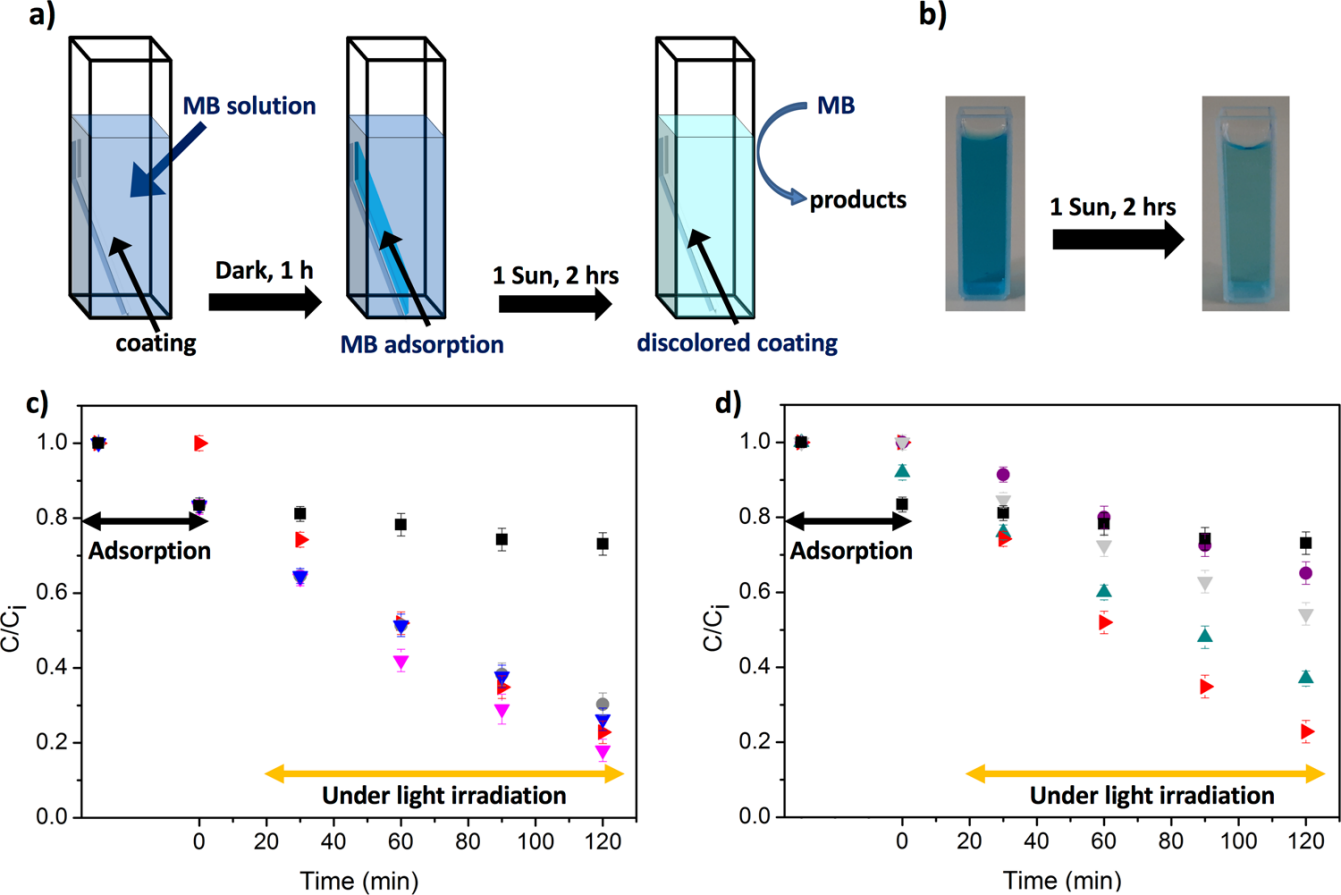
(a) Scheme of the photodegradation of aqueous MB (3 mL, 25 μM) by the composite materials. The coatings were soaked into a quartz cuvette containing the MB solution and kept in the dark for 1 h to allow for the dye adsorption. Then, the quartz cuvette was irradiated using a solar simulator at 1 sun allowing for the discoloration of the MB solution by the soaked composite coatings. (b) Picture of the samples where the result of a typical photocatalytic cycle resulting in the discoloration of the MB solution is evident. (c) Decrease in MB concentration vs time in samples containing HC and (d) CC systems. In both cases, adsorption–desorption of the dye occurs in about 1 h, and it is different between HC and CC samples. MB photodegradation values are reported at different TiO_2_ loadings for HC systems, 5 (gray circles), 15 (blue triangles), and 35 wt % (magenta triangles), and similarly for CC systems, 5 (purple circles), 15 (light gray triangles), and 35 wt % (green triangles). Data for bare PDHS-POTS (black squares) and TiO_2_ (red triangles) are reported in both panels.

In Figure 6c,d, we report the analysis of photocatalytic degradation of the MB solution obtained from absorption spectra of the MB solution (see Figure S4) containing HC and CC nanocomposites acquired at defined time intervals after irradiation at 1 sun. In Figure 6a,b, the measurement steps are sketched. The two systems were soaked into a quartz cuvette containing the MB solution at the initial concentration (*C*_i_) equal to 2.5 × 10^–5^ M and kept in the dark to allow for the dye adsorption until adsorption–desorption equilibrium is reached in about 1 h. The photocatalytic activity results in discoloration of the MB solution, evident by eye inspection and quantitatively evaluated by monitoring the absorbance intensity at 664 nm (Figure 6b).

As shown in Figure 6a,b, MB adsorption in the dark is well-observed in all the HC systems, reaching efficiencies in 60 min at ambient conditions, similar to the corresponding one in the PDHS-POTS. On the contrary, the adsorption performance in the dark is less evidently efficient for the CC system (especially for TiO_2_ loadings at 5 or 15 wt %). Only in the case of the TiO_2_ loading equal to 35 wt %, a small adsorption performance in 1 h is observed. Under solar irradiation, the addition of TiO_2_ nanoparticles to the polymer increases the MB degradation in both HC and CC systems. As can be seen, different behaviors are found in HC and CC samples. Both samples present a photocatalytic activity. CC samples present a behavior strictly dependent on the amount of loaded TiO_2_ nanostructures. This evidence can be explained according to the different TiO_2_ cluster morphology and surface chemistry of HC and CC systems. For the HC composite, the increase in the TiO_2_ loading does not significantly modify MB adsorption. In fact, the good MB photodegradation efficiency due to the formation of TiO_2_ clusters is already observed at a 5 wt % loading. Differently, the CC composite, characterized by a lower MB adsorption and lower TiO_2_ cluster formation, resulted in a good MB photodegradation only at a 35 wt % TiO_2_ loading. Importantly, by taking constant the TiO_2_ loading in the composites, the measurements highlight that the HC system produces a more efficient MB photodegradation in comparison to the CC system. A degradation efficiency of about 80% was obtained for the HC at a 35 wt % TiO_2_ loading. The observed photodegradation value is comparable to that of TiO_2_ coating and higher than the one observed for the CC system at the same TiO_2_ loading, which is equal to 63%. This improvement of the HC systems can be ascribed to the realization of a structural architecture in the composite favorable to the degradation of the dye, thus accounting for the improved HC adsorption performance than that measured in CC. The dye under solar irradiation interacts in bulk with larger TiO_2_ clusters formed after the thermal treatment. The larger size and the good interconnection between TiO_2_ in the cluster, also leading to TiO_2_ nanoparticle sintering,^48,49^ could improve the charge separation and transport of the light-generated carriers to reach the catalyst and interact with H_2_O or O_2_ molecules.^36,38^ This effect could be in turn ascribed to the higher time of diffusion and the lower recombination of the light-generated carriers within the larger TiO_2_ cluster.^38,50^ The photocatalytic degradation rate follows pseudo-first-order kinetics (Figure S5). The apparent rate constant (*k*) was evaluated by the linear regression model expressed by ln(*C*/*C*_0_) = *kt*, where *C*_0_ is the concentration after adsorption–desorption equilibrium and *C* is the concentration after a given exposure at 1 sun irradiation time of the MB aqueous solution. As expected, the apparent reaction constant *k* increases as a function of the TiO_2_ loading for both CC and HC systems (Table S1). The apparent reaction constants *k* for PDHS-POTS/TiO_2_ (35 wt %) HC and PDHS-POTS/TiO_2_ (35 wt %) CC were 0.013 and 0.007 min^–1^, respectively, the *k* value for the HC system being comparable with the corresponding one of the TiO_2_ coating (0.012 min^–1^). The values of *k* for photocatalytic degradation of the MB dye by PDHS-POTS/TiO_2_ (35 wt %) HC coatings were similar to previous reports, although different experimental conditions were employed, in particular considering the TiO_2_ nanoparticle/polymer combinations and the light irradiation conditions. Table S2 reports a comparison with previous MB TiO_2_-catalyzed photodegradation studies. Similarly to nanoparticles, also, 1D TiO_2_ nanomaterials have been used to obtain composites with polymers, e.g., with polydimethylsiloxane, resulting in excellent mechanical stability and good photocatalytic properties toward MB photodegradation.^32^ Some studies have also focused on the preparation of nanocomposites containing Au nanostructures with amorphous titania^51,52^ or lanthanide-doped nanophosphors covered by porous TiO_2_ and Ag–Cu nanoparticles,^53^ the latter permitting absorption of photons from the ultraviolet to the near infrared region, resulting in the MB degradation rate up to 0.028 min^–1^ under UV light illumination.^53^ Noteworthy, the dye degradation kinetics observed in the HC and CC systems was similar to the one catalyzed by P25 TiO_2_ nanoparticle composites in porous PMDS materials^28^ or in fluorinated polysiloxanes^29^ or by TiO_2_ doped with sulfur.^54^ As far as the degradation of MB by photocatalysis in aqueous systems is concerned, this method allows obtaining less harmful products with respect to the MB itself,^55^ through the formation of aromatic reaction intermediates, finally resulting in a quasi-complete mineralization of the C, N, and S elements into CO_2_, NH_4_^+^, NO_3_^–^, and SO_4_^2–^.^56^ In particular, the mechanism of MB degradation in aqueous environments by TiO_2_-based heterogeneous photocatalysis has been thoroughly investigated by Houas et al.^57^ Briefly, MB decomposition is due to cascade oxidation reactions triggered by the oxidizing species OH^·^ in turn resulting from the holes and electrons produced from the photons absorbed by titania.^58^ The authors evaluated the degradation reactions due to hydroxylation and aromatic ring opening by gas chromatography–mass spectrometry analyses, ultimately observing a quasi-complete mineralization of the C, N, and S elements into CO_2_, NH_4_^+^, NO_3_^–^, and SO_4_^2–^. Indeed, the photodegradation products are significantly less toxic than MB, therefore permitting titania-based heterogeneous photocatalysis to be ideally suited for the degradation of MB-contaminated wastewater.^57^

The photocatalytic reusability of the PDHS-POTS HC and the CC systems was tested by repeating the photocatalytic cycles up to three times. In between each photocatalytic test, the sample was washed with ultrapure water. The results highlighted significant differences between the two cases. In the HC system, a small loss of efficiency (from 79 to 72% and finally 64%) is noted only at the third degradation cycle. Interestingly, the HC is partially discolored after each cycle of photocatalysis (see Figure S6). Differently, in the case of CC, the loss of efficiency from the first to the third cycle (from 63 to 53% and finally 33%) is significantly higher, and the discoloration is significantly lower after each photocatalysis cycle, ultimately resulting in a lower reusability in comparison to HC. The better reusability performance of the HC system could be ascribed to the higher surface hydrophilicity in comparison to the corresponding CC, which in turn permits easier removal of the adsorbed MB molecules by simple immersion in ultrapure water. The MB removal efficiency shows an opposite trend vs the surface hydrophilicity tuned by the TiO_2_ loading in the HC and CC systems. For both HC and CC, the TiO_2_ loading leads to an increase in MB removal. While in HC, the increase in the TiO_2_ loading leads to a slight decrease in the surface hydrophobicity, the opposite is observed in the CC system since the increase in the TiO_2_ loading increases hydrophobicity. This difference stems from the largely distinct architectures of the two composites. Finally, the reproducibility of the PDHS-POTS HC at 35 wt %TiO_2_ was tested by replicated photocatalysis experiments on three different samples (see Figure S7), resulting in an excellent reproducibility of the coating photocatalytic activity.

### 3.5 Discussion of the Evaporation Speed Effects

The morphological investigations of the TiO_2_ nanoparticle composites with PDHS-POTS highlight a significantly different scenario between the HC and the CC systems. The wettability and organization of the TiO_2_ clusters within the polymeric matrix are a consequence of the different solvent evaporation dynamics of the two systems, ultimately resulting in a two-fold increase in the photocatalytic activity of HC with respect to CC. In general, the CC systems show a highly hydrophobic behavior, with TiO_2_ clusters almost uniformly dispersed among the surface and the bulk of the polymeric matrix, the diffusion effects being dominant over the evaporation rate (i.e., *Pe* < 1). On the other hand, the HC systems show a more hydrophilic surface than the bare PDHS-POTS system likely due to the weak segregation of high-surface energy TiO_2_ aggregates with sizes significantly smaller than those of bulk clusters. This is due to the higher evaporation rate, which overcomes diffusion phenomena (i.e., *Pe* > 1). Differently from CC, the bulk TiO_2_ clusters have a significantly larger size than the surface-segregated clusters, as shown by SEM images of the top surfaces and bulk CLSM imaging. Such a small-on-top-on architecture might seem counterintuitive since larger particles should have a smaller diffusion constant and a higher *Pe* and should remain closer to the drying interface. However, the here observed small-on-top scenario observed in the experiments agrees well with theoretical models developed under different approaches, which all describe well the distribution of colloids of two different sizes at *Pe* > 1. The approach from Fortini et al.^59^ demonstrated that a rapidly drying interface (*Pe* > 1) causes an osmotic pressure that pushes larger particles away from it much faster than smaller particles. The result is the formation of an out-of-equilibrium small-on-top-on architecture. This study was extended by Howard et al.^60^ adopting dynamic density functional theory, finding the same stratification type even at lower *Pe*. Zhou and coworkers^61^ further generalized this study by considering the interaction between differently sized particles at different *Pe*, corroborating the findings from Fortini et al.^59^ and further expanding this dynamics at different regimes of particles sizes and drying rates. The emergence of such a small-on-top condition is observed if
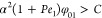
where α can be defined as the size ratio of the bigger particles to the smaller ones: *r*_2_/*r*_1_ > 1, *Pe*_1_ is the *Pe* of smaller particles, φ_01_ is the number density of the smaller particles, and *C* is a fitting parameter. The size ratio plays a fundamental role in selecting this out-of-equilibrium phenomenon: the higher is the α, the higher will be the small-on-top stratification. The emerging question is why the TiO_2_ clusters are formed within the polymeric matrix in the two different scenarios, CC and HC. This can be explained by considering the effect caused by temperature in promoting nanoparticle clustering in the HC system due to solvent evaporation.^25^ It has been demonstrated both theoretically^62^ and experimentally^63,64^ that the particle/particle and particle/polymer interactions play a fundamental role in the observed size of the aggregated clusters, during the drying dynamics within the polymer composite. Our observations are well in accord with the molecular dynamics simulations from Cheng and Grest^65^ who found that in the case of weak particle/polymer interaction, the particles would distribute in the bulk of the polymer and partially at the drying interface. In turn, the formation of particle clusters within the polymer matrix is facilitated by charge-stabilized particles and low affinity between particles and the polymer, leading to clusters if particle/particle interaction is good and particle/polymer interaction is weak. Differently, if the particle/particle interaction becomes weak due to charge screening (e.g., high pH^63^), then the particles do not cluster and a higher dispersion with the polymer matrix will be observed. By considering that EtOAc is an extremely weak basic system (p*K*_a_ around 25) and that the isoelectric point of the P25 TiO_2_ nanoparticles is approximately 6.2,^66^ these are expected to cluster during solvent drying, as no charge-induced screening of repulsive interactions might take place. Finally, the apparently globular morphology of the observed clusters could be ascribed to the lack in structural ordering from the obtained polymer matrix, in accordance with the observations from Chen et al.^67^ who demonstrated both through numerical simulations and experiments that only in the presence of a nematically ordered polymer, anisotropic particle aggregates evolving in highly connected structures could be obtained upon solvent evaporation. A polymeric system lacking the possibility to form ordered domains produces globular clusters, as is in our experiments. Indeed, the obtained globular clusters show similar morphology to the ones formed from bare TiO_2_ films (i.e., in the absence of the polymer).

**Figure 7:**
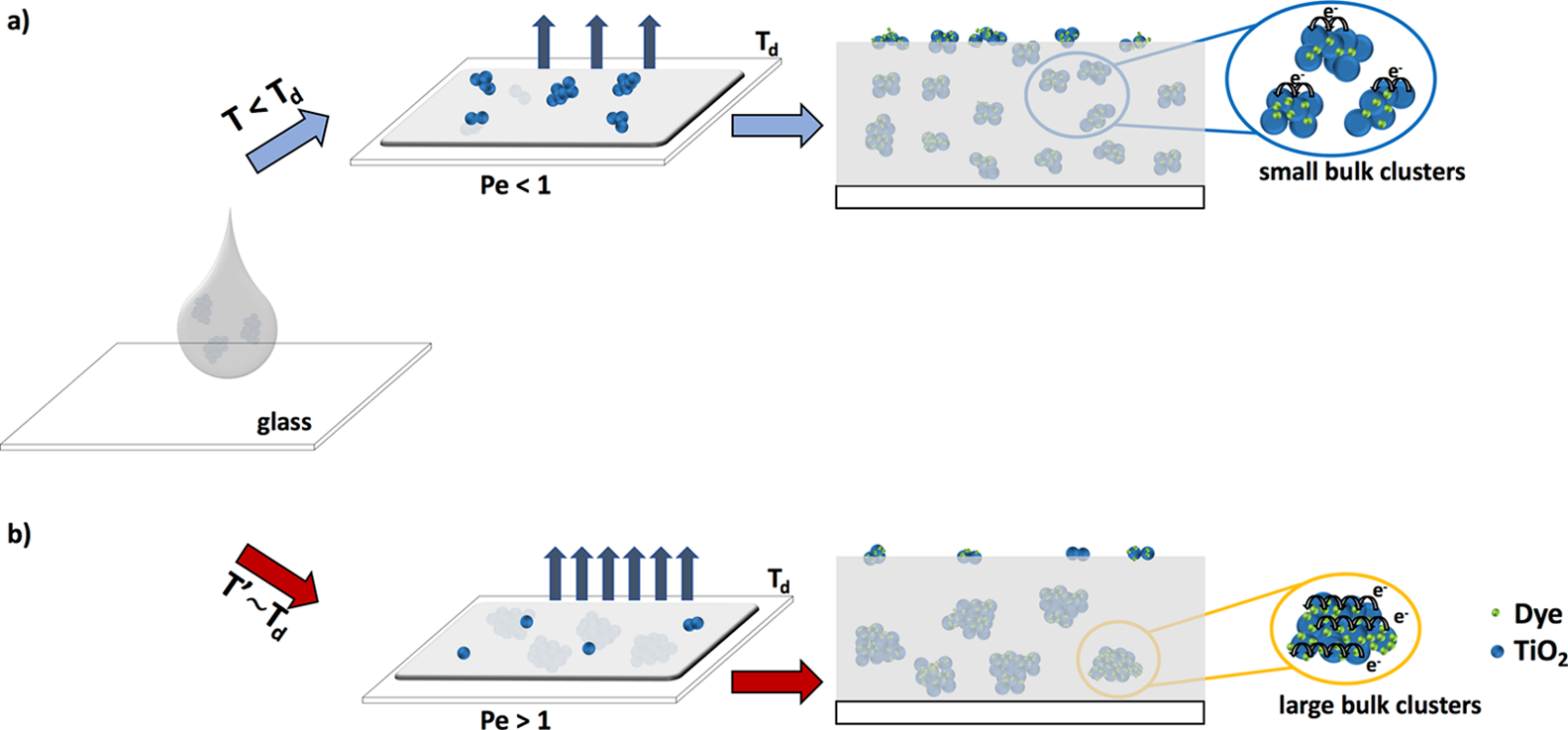
Tailoring evaporative speed leads to reconfigurable TiO_2_ nanoparticle bulk clustering in polysiloxane matrices. (a) Depositing the coating at temperatures below the solvent evaporation temperature (*T* < *T*_d_), the evaporative speed will be low (i.e., low *Pe*), resulting in uniform surface segregation/bulk dispersion of TiO_2_ nanoparticle clusters. (b) Depositing the coating at temperatures close to the solvent evaporation temperature (*T*′ ∼ *T*_d_), the evaporative speed will be high (i.e., high *Pe*), resulting in surface segregation of small TiO_2_ nanoparticle clusters and bulk dispersion of larger TiO_2_ nanoparticle clusters.

The increase in the polymer/nanoparticle mixture evaporation rate (i.e., *Pe* > 1) permits acceleration of the nanoparticle clustering effect by more rapidly removing solvent molecules from the system^67^ since the entropy of the polymer molecules is maximized if TiO_2_ nanoparticles form clusters as they decrease their exposure to the polymer molecules. As shown in this work, the bulk clustering of larger TiO_2_ nanoparticles in the HC leads to a 2-fold increase in the photocatalytic activity with respect to CC (see Figure 7), as a consequence of the interplay of higher MB adsorption and higher photocatalytic efficiency of the bulk segregated clusters of the HC system.

## 4 Conclusions

The mixing control of nanoparticles within polymer matrices is an emergent approach for the tailored preparation of composite materials. It is well-known that by selecting out-of-equilibrium conditions during the assembly, it is possible to enlarge the landscape of new structures resulting in reconfigurable physical–chemical properties. This study has focused on the effect of temperature on the preparation of nanoparticle/polymer composites, i.e., heating the sol at the evaporation temperature of the solvent, toward the control of the surface segregation/bulk dispersion of photoactive TiO_2_ nanoparticle clusters into fluorinated polysiloxanes.

In the absence of thermal treatments, the nanoparticle aggregates covered by polymer molecules are formed at the water/air interface, finally resulting in a superhydrophobic surface with discrete photocatalytic properties. The thermally induced bulk sintering of large TiO_2_ nanoparticle clusters and the formation of smaller hydrophilic TiO_2_ nanoparticle clusters lead to surface properties similar to those of the bare polymer. In turn, this allowed us to maximize the adsorption phenomena and the diffusion time of the carriers on the TiO_2_ nanoparticles in the bulk clusters. These synergic effects resulted in a two-fold increase in the MB photodegradation efficiency, higher reusability with respect to the system produced without thermal treatments, and even MB photodegradation efficiency similar to that of the bare TiO_2_ coating. These results pave the way toward new thermally guided pathways enabling the engineering of polymer/nanoparticle composites merging the physicochemical properties of both nanoparticles and polymer systems ideally and becoming an excellent toolbox for exploring different polymer/nanoparticle combinations.

## Supporting Information Available

SEM images of the TiO_2_ nanoparticle clusters, automated grain segmentation analysis, estimation of average roughness analysis from SEM images, UV–vis spectra of MB solution, kinetic curves of MB degradation of the HC and CC systems, photograph of the HC and the CC systems, apparent rate constants of MB photodegradation under 1 sun illumination, summary of TiO_2_-catalyzed MB photodegradation efficiencies, and reproducibility tests (PDF)
