# Supplementary material for: Improved Photocatalytic Activity of Polysiloxane TiO2 Composites by Thermally Induced Nanoparticle Bulk Clustering and Dye Adsorption
Source: Langmuir. 2021 Aug 17;37(34):10354–65. doi: 10.1021/acs.langmuir.1c01475 (PMC8413002; doi:10.1021/acs.langmuir.1c01475)
Supplement: Supplementary file 1 — la1c01475_si_001.pdf [file la1c01475_si_001.pdf]

SUPPORTING INFORMATION

Improved Photocatalytic Activity of Polysiloxane TiO_2 Composites by thermal-induced nanoparticle Bulk Clustering and Dye Adsorption

*Clara Chiappara,[†,//] Giuseppe Arrabito,[†] Vittorio Ferrara,[//,§] Michelangelo Scopelliti,[†] Giuseppe Sancataldo,[†] Valeria Vetri,[†] Delia Francesca Chillura Martino[//,§] * and Bruno Pignataro[†,//]**

[†]Department of Physics and Chemistry (DiFC) Emilio Segrè, University of Palermo, Building 17, V.le delle Scienze, Palermo 90128, Italy.

[//]National Interuniversity Consortium of Materials Science and Technology (INSTM), UdR of Palermo, 50121 Florence, Italy.

[§]Department of Biological, Chemical and Pharmaceutical Sciences and Technologies (STEBICEF), University of Palermo, Building 16, V.le delle Scienze, Palermo 90128, Italy.

*To whom correspondence should be addressed. E-mail: delia.chilluramartino@unipa.it bruno.pignataro@unipa.it

Table of Contents:

Figure S1. SEM images of the TiO_2 nanoparticles clusters.

Figure S2. Automated grain segmentation analysis.

Figure S3. Estimation of average roughness analysis from SEM images.

Figure S4. UV-vis spectra of MB solution.

Figure S5. Kinetic curves of MB degradation of the HC and CC system.

Figure S6. Photograph of (a) the HC system and (b) the CC system.

Table S1. Apparent rate constants (k) of MB photodegradation under 1 Sun illumination.

Table S2. Summary of TiO_2 catalyzed MB photodegradation efficiencies.

Figure S7. Reproducibility tests.

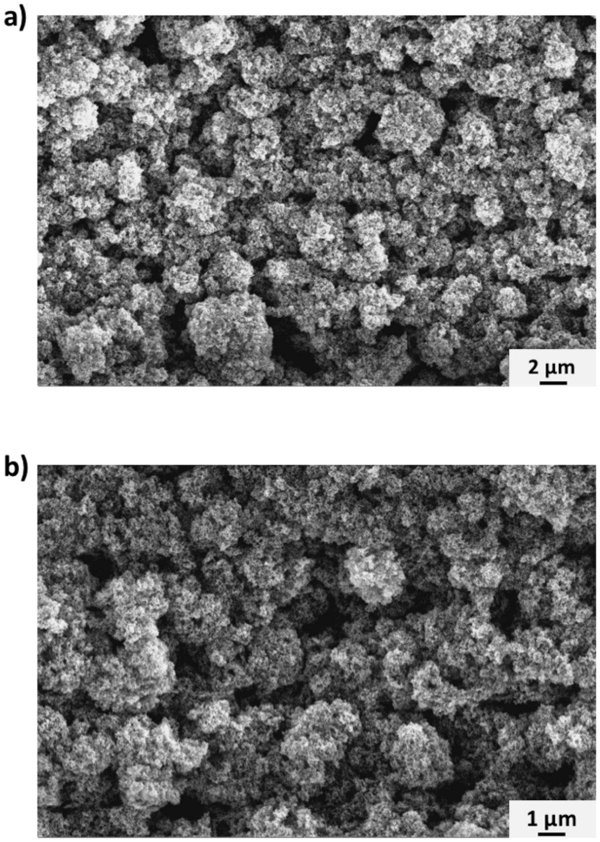

Figure S1. SEM images of the TiO_2 nanoparticles forming clusters with globular morphology at (a) 10,000 magnification and (b) 20,000 magnification.

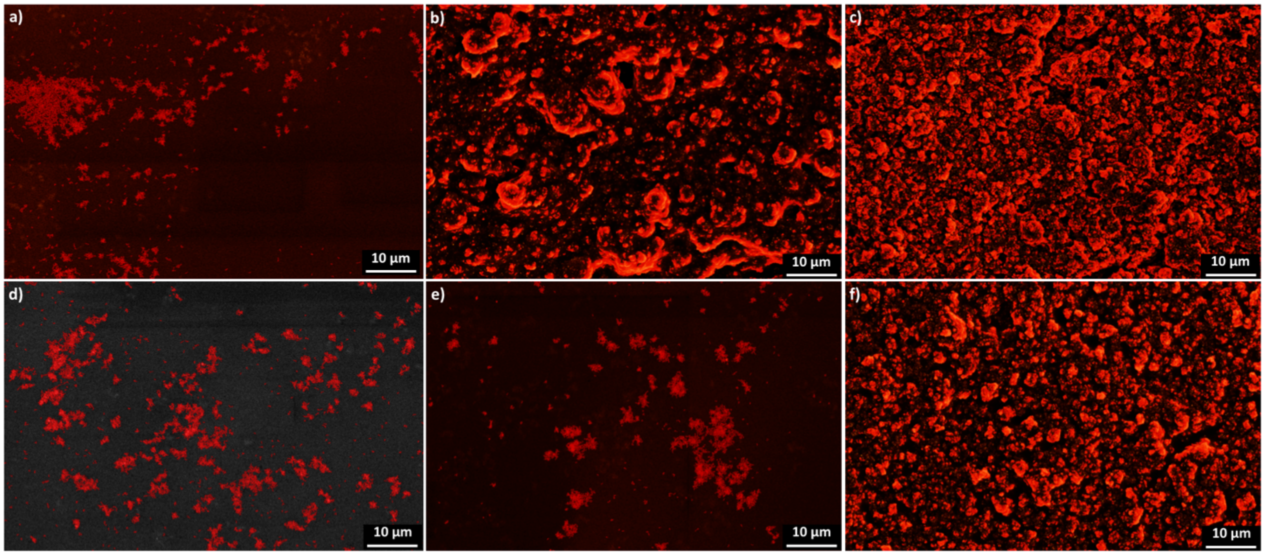

Figure S2. Automated grain segmentation analysis on **a)** PDHS-POTS/TiO_2 (5 wt %) CC, **b)** PDHS-POTS/TiO_2 (15 wt %) CC, **b)** PDHS-POTS/TiO_2 (35 wt%) CC, **d)** PDHS-POTS/TiO_2 (5 wt %) HC, **e)** PDHS-POTS/TiO_2 (15 wt %) HC and **f)** PDHS-POTS/TiO_2 (35 wt %) HC.

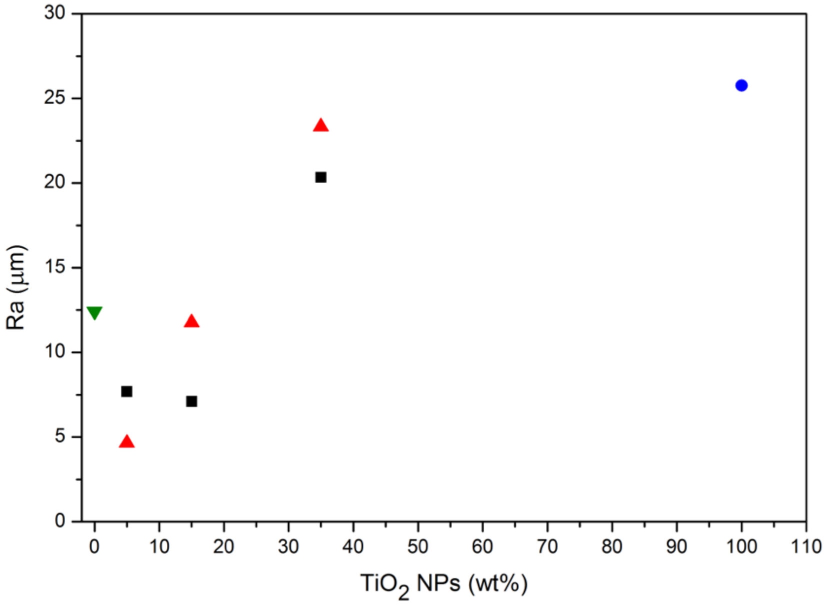

Figure S3. Estimation of average roughness analysis on SEM images on HC (red triangles) and CC (black squares) as a function of TiO_2 loading. As per reference, the roughness values for the bare PDHS-POTS (green triangle) and TiO_2 coatings (blue circle) are reported.

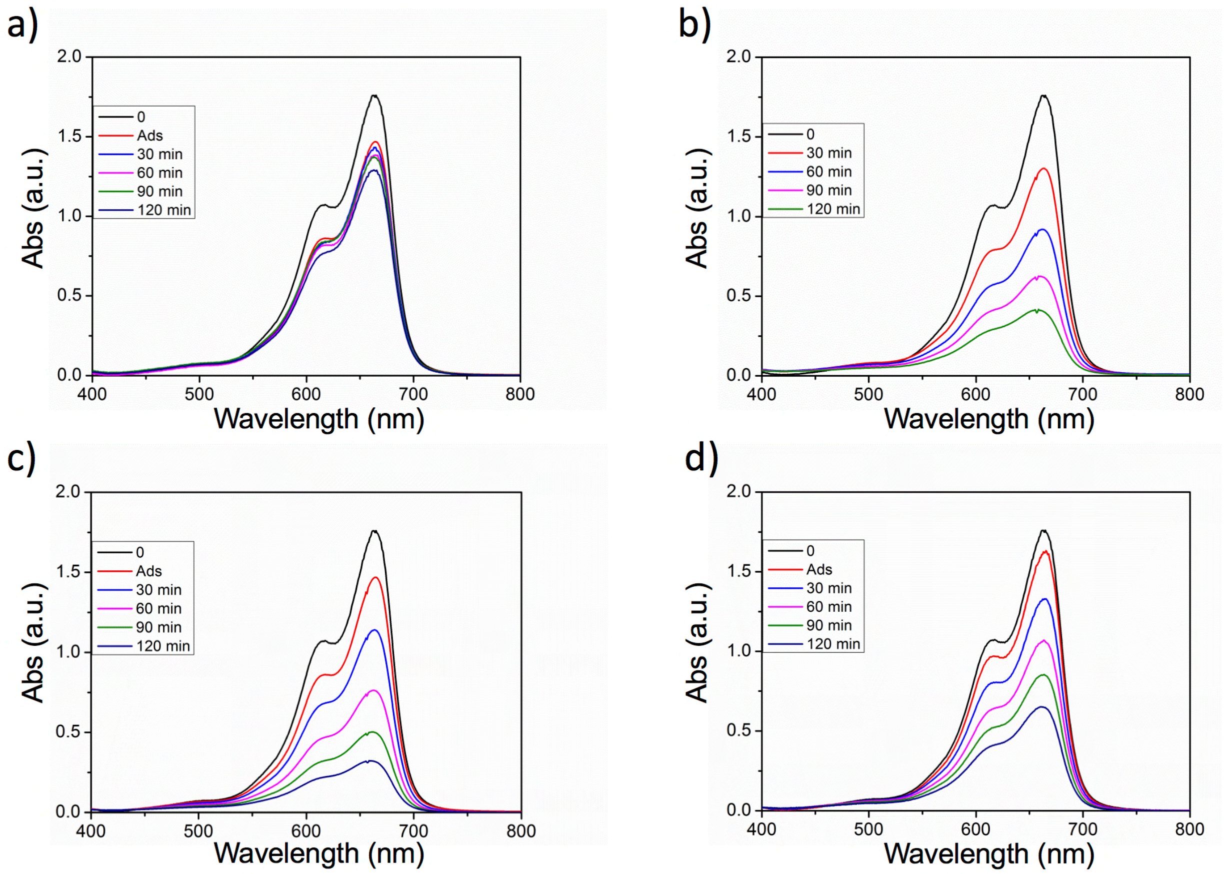

Figure S4. UV-vis spectra of MB solution after treatment with **(a)** PDHS-POTS coating, **(b)** TiO_2 P25 coating, **(c)** HC and **(d)** CC coatings.

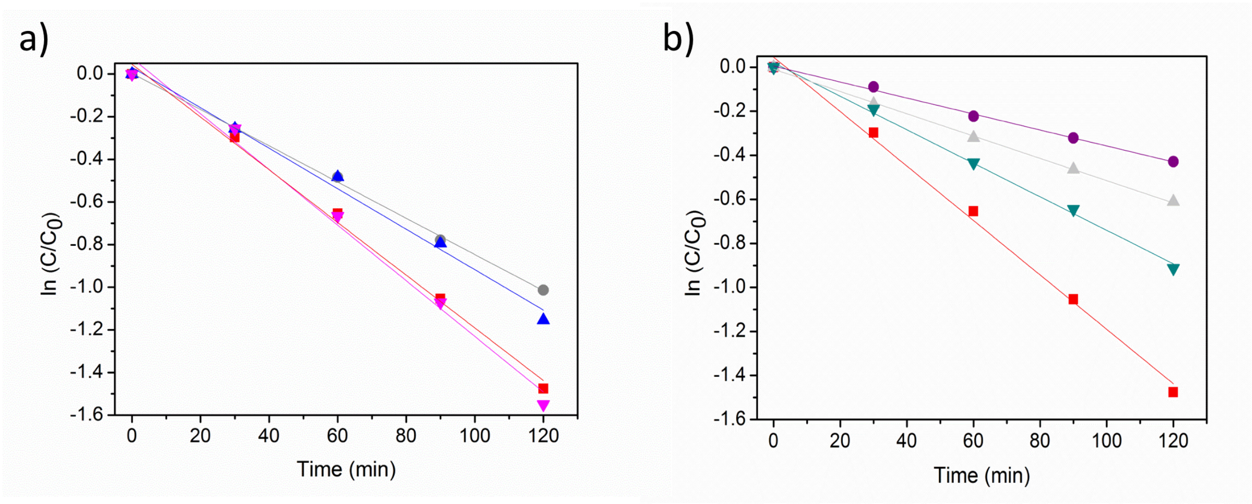

Figure S5. Kinetic curves of MB degradation under 1 Sun obtained for the HC systems at different TiO_2 loadings **(a)** - 5 wt % (grey circles), 15 wt% (blue triangles), 35 wt % (magenta triangle) - and for the CC systems at different TiO_2 loadings **(b)** - 5 wt % (purple circles), 15 wt % (light grey triangles) and 35 wt % (green triangles). The kinetic curve for MB degradation from bare TiO_2 (red squares) is reported for comparison.

**Coatings**	**k (CC)**	**k (HC)**
PDHS-POTS/TiO_2 (5 wt%)	0.0036 ± 0.0001	0.0085 ± 0.0002
PDHS-POTS/TiO_2 (15 wt%)	0.0051 ± 0.0001	0.0095 ± 0.0005
PDHS-POTS/TiO_2 (35 wt%)	0.0076 ± 0.0002	0.013 ± 0.001

Table S1. Apparent rate constants (expressed in min^{-1}) of MB solution degradation under 1 Sun irradiation from the CC and HC systems as a function of the TiO_2 loading.

Photocatalyst	Light source, intensity	Dark absorption time (min)	Degradation time (min)	% Degradation	Apparent rate constant (min^{-1})	Bibliographic reference
**TiO_2/reduced graphene oxide nanocomposite**	Visible light (50 W Xe lamp)	30	150	96	0.0039	1
**TiO_2/graphene oxide**	Solar simulator (300W Xe lamp PLS-SXE300)	30	120	95	0.0141	2
**TiO_2/kaolin nanocomposite**	Visible light (70W Philips halogen lamps)	30	270	90	n.r.	3
**TiO_2/zeolite nanocomposite**	UV light (125 W 365 nm)	30	60	93	0.04694	4
**TiO_2/silica**	UV light (30 W 366 nm Philips UV lamp)	15	40	99	0.003741	5
**TiO_2/ reduced graphene oxide nanocomposite**	UV light (16 W 254 nm UV light lamp)	30	60	99	0.0678	6
**TiO_2/Poly-N-isopropylacrylamide**	Hg lamp (7.13 W 365 nm)	30	210	86	n.r.	7
**TiO_2/reduced graphene oxide/ Poly(methyl methacrylate) nanocomposite**	UV light (15 W UV-C lamp)	10	120	90	0.0434	8
**TiO_2/graphene oxide/ polyacrylamide**	Solar simulator (150 W Xe Arc lamp, 10500, Abet Technolgies)	30	90	>99	0.0276	9
**TiO_2/graphene oxide/Polyaniline nanocomposite**	UV light (power not reported)	/	60	98	n.r.	10
**Sulfur doped TiO_2 films**	Solar simulator (TS Space Systems solar)	60	~180	~80	n.r.	11
**Multi doped Brookite TiO_2**	Visible light (150 W Xe-lamp ORIEL)	30	150	~100	0.041	12
**TiO_2 Degussa P25**	Visible light (150 W Xe-lamp ORIEL)	30	1200	~100	0.005	12
**$NaYF_4$:Yb/Tm@N**	UV light (8 W);	30	60	96 (UV light);	0.0028; 0.00045;	13

**aYF_4:Yb nanoparticles coated with TiO_2 shell and Ag-Cu nanoparticles**	Visible light (45 W); NIR light (OAI Trisol solar simulator, filtered for NIR)			60 (visible light); 65 (NIR light)	0.0009	

Table S2. Summary of TiO_2 catalyzed MB photodegradation efficiencies in the literature. The reports are categorized according to the light source, intensity, dark absorption time (min), experimental time for the degradation (min), percentage degradation and apparent rate constant (min^{-1}).

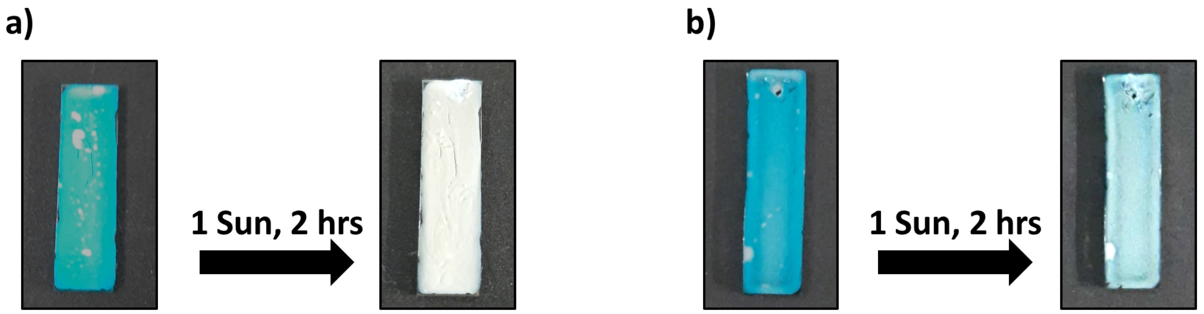

Figure S6. Photograph of **(a)** the HC system and **(b)** the CC system immediately after MB adsorption (figure on the left in each panel) and after one photocatalysis cycle (figure on the right in each panel). The HC system shows a much more efficient discolouration after each photocatalysis cycle.

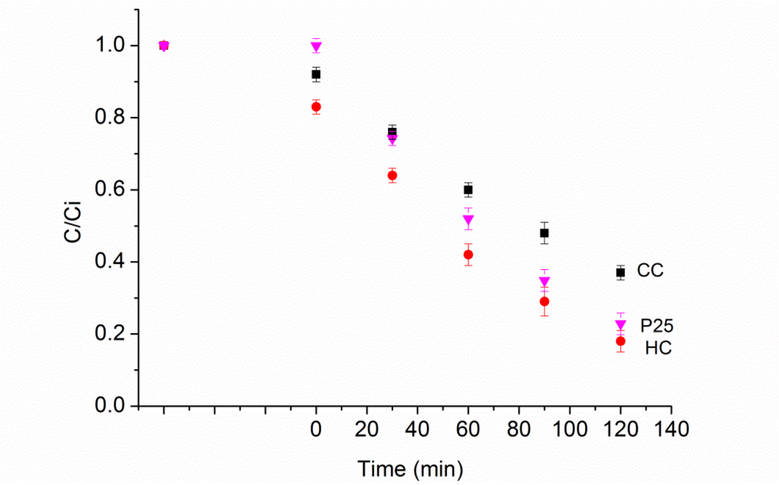

Figure S7. Photocatalytic degradation of MB aqueous solution on PDHS-POTS/TiO_2 (35 wt %) in the HC (red circles), CC (black squares) and TiO_2 P25 (magenta triangles) coating. The reported error bars are calculated on three different samples at each time of photoreaction.

Bibliography

(1) Yang, Y.; Wang, W.; Li, H.; Jin, X.; Wang, H.; Zhang, L.; Zhang, Y. NH2-MIL-53(Al) Nanocrystals Anchored on the Surface of RGO Hollow Spheres and Its Visible Light Degradation of Methylene Blue. *Mater. Lett.* **2017**, *197*, 17–20. https://doi.org/10.1016/j.matlet.2017.03.041.

(2) Wang, M.; Cai, L.; Jin, Q.; Zhang, H.; Fang, S.; Qu, X.; Zhang, Z.; Zhang, Q. One-Pot Composite Synthesis of Three-Dimensional Graphene Oxide/Poly(Vinyl Alcohol)/TiO2 Microspheres for Organic Dye Removal. *Sep. Purif. Technol.* **2017**, *172*, 217–226. https://doi.org/10.1016/j.seppur.2016.08.015.

(3) Wongso, V.; Chen, C. J.; Razzaq, A.; Kamal, N. A.; Sambudi, N. S. Hybrid Kaolin/TiO2 Composite: Effect of Urea Addition towards an Efficient Photocatalyst for Dye Abatement under Visible Light Irradiation. *Appl. Clay Sci.* **2019**, *180*, 105158. https://doi.org/10.1016/j.clay.2019.105158.

(4) Liao, G.; He, W.; He, Y. Investigation of Microstructure and Photocatalytic Performance of a Modified Zeolite Supported Nanocrystal Tio2 Composite. *Catalysts* **2019**, *9*, 502. https://doi.org/10.3390/catal9060502.

(5) Fatimah, I.; Prakoso, N. I.; Sahroni, I.; Musawwa, M. M.; Sim, Y. L.; Kooli, F.; Muraza, O. Physicochemical Characteristics and Photocatalytic Performance of TiO2/SiO2 Catalyst Synthesized Using Biogenic Silica from Bamboo Leaves. *Heliyon* **2019**, *5*, e02766. https://doi.org/10.1016/j.heliyon.2019.e02766.

(6) Mohamed, H. H.; Alsanea, A. A. TiO2/Carbon Dots Decorated Reduced Graphene Oxide Composites from Waste Car Bumper and TiO2 Nanoparticles for Photocatalytic Applications. *Arab. J. Chem.* **2020**, *13*, 3082–3091. https://doi.org/10.1016/j.arabjc.2018.08.016.

(7) Duan, Y.; Ma, J.; Liu, J.; Qiang, L.; Xue, J. Facile Synthesis of Thermo-Responsive TiO2/PNIPAM Composite with Switchable Photocatalytic Performance. *Fibers Polym.* **2020**, *21*, 717–723. https://doi.org/10.1007/s12221-020-9724-z.

(8) Torabi Momen, M.; Piri, F.; Karimian, R. Photocatalytic Degradation of Rhodamine B and Methylene Blue by Electrochemically Prepared Nano Titanium Dioxide/Reduced Graphene Oxide/Poly (Methyl Methacrylate) Nanocomposite. *React. Kinet. Mech. Catal.* **2020**, *129*, 1145–1157. https://doi.org/10.1007/s11144-020-01722-x.

(9) Moztahida, M.; Lee, D. S. Photocatalytic Degradation of Methylene Blue with P25/Graphene/Polyacrylamide Hydrogels: Optimization Using Response Surface Methodology. *J. Hazard. Mater.* **2020**, *400*, 123314. https://doi.org/10.1016/j.jhazmat.2020.123314.

(10) Baruah, S.; Kumar, S.; Nayak, B.; Puzari, A. Optoelectronically Suitable Graphene Oxide-Decorated Titanium Oxide/Polyaniline Hybrid Nanocomposites and Their Enhanced Photocatalytic Activity with Methylene Blue and Rhodamine B Dye. *Polym. Bull.* **2021**, *78*, 1703–1720. https://doi.org/10.1007/s00289-020-03182-8.

(11) Xiong, Y.; He, D.; Jaber, R.; Cameron, P. J.; Edler, K. J. Sulfur-Doped Cubic Mesostructured Titania Films for Use as a Solar Photocatalyst. *J. Phys. Chem. C* **2017**, *121*, 9929–9937. https://doi.org/10.1021/acs.jpcc.7b01615.

(12) Cataldo, S.; Weckhuysen, B. M.; Pettignano, A.; Pignataro, B. Multi-Doped Brookite-Prevalent TiO2 Photocatalyst with Enhanced Activity in the Visible Light. *Catal. Letters*

2018, *148*, 2459–2471. https://doi.org/10.1007/s10562-018-2463-8.

(13) Reddy, K. L.; Kumar, S.; Kumar, A.; Krishnan, V. Wide Spectrum Photocatalytic Activity in Lanthanide-Doped Upconversion Nanophosphors Coated with Porous TiO2 and Ag-Cu Bimetallic Nanoparticles. *J. Hazard. Mater.* **2019**, *367*, 694–705. https://doi.org/10.1016/j.jhazmat.2019.01.004.
